# Biomass production and removal of ammonium and phosphate by *Chlorella* sp. in sludge liquor at natural light and different levels of temperature control

**DOI:** 10.1186/s40064-016-2266-6

**Published:** 2016-05-23

**Authors:** Anette M. Åkerström, Leiv M. Mortensen, Bjørn Rusten, Hans Ragnar Gislerød

**Affiliations:** Department of Plant Sciences, Norwegian University of Life Sciences, P.O. Box 5003, 1432 Ås, Norway; Aquateam COWI AS, Rodelokka, P.O. Box 6875, 0504 Oslo, Norway

**Keywords:** Microalgae, Temperature, Sludge liquor, Biomass, Natural light

## Abstract

Microalgae cultivation for biomass production and nutrient removal implies the use of natural light and minimal control of the temperature for obtaining a low cost production. The aim of this study was to quantify the effect of temperature control at natural light on biomass productivity and removal of NH_4_-N and PO_4_-P of a mesophilic strain of *Chlorella. Chlorella* sp. was grown in reject water of anaerobically digested municipal sludge, sludge liquor, inside a greenhouse compartment (Ås, Norway, 59°N) using batch cultures (300 mL). Five experiments were conducted from May to September, and effects of different levels of temperature control and diurnal variations were investigated. The highest biomass productivities (0.45 g L^−1^ day^−1^) in the linear growth phase were obtained at daily light integrals ≥12 mol day^−1^ m^−2^. Results showed that the average temperature was of more importance than the night or day temperature range. At average temperatures <22 °C for cultures with no temperature control, the productivity decreased by 23 and 39 % compared to cultures with full temperature control (24–25 °C). In one experiment, the productivity was reduced at no temperature control due to prolonged high daytime temperatures (>32 °C) and were followed by a lower NH_4_-N removal rate. Otherwise, temperature had little effect on NH_4_-N removal. The level of temperature control did not affect removal of PO_4_-P. Cellular starch content varied from ~15–38 % in the evening and was generally lower at no temperature control. In the morning the starch content was reduced to ~4–12 % with no difference between the different levels of temperature control. (~4–12 %).

## Background

Microalgae cultivation for biomass production has a higher environmental impact when considering energy use, greenhouse gas emissions and water usage, compared to conventional crops (Clarens et al. 2010). The environmental impact can only be reduced by using wastewater and flue gas as a nutrient and carbon source (Clarens et al. 2010). Sludge liquor (SL) produced in the dewatering process of anaerobically digested sludge has proven to be a suitable substrate for algal cultivation with the dual effects of nutrient removal and biomass production (Rusten and Sahu 2011; Yuan et al. 2012; Åkerström et al. 2014). Integrating this process at a wastewater treatment plant (WWTP) requires minimal input of energy to make the process economically and environmentally viable. This implies the use of only solar radiation that is an ever-changing factor. Temperature is another key parameter in algae cultivation (Ras et al. 2013) and “temperature control needs to be considered when evaluating the technical feasibility and costs in algae production” (Bechet et al. 2010). By using existing infrastructure at WWTPs, algae biomass can be anaerobically digested and the main retro-fitting unit would be the bioreactor (Sahu et al. 2013).

Irradiance is the main environmental parameter in autotrophic algae cultivation. SL is highly turbid due to a high content of suspended solids (SS) that reduces light availability for photosynthesis. Although microalgae can grow in raw SL, the growth rate is significantly increased when lowering the content of SS, which can be carried out by the simple process of dilution (Åkerström et al. 2014).

At high latitudes such as Norway, the growth season spans from April to September. During that period, the photoperiod changes from 8 to 16 h. Within the day, the photosynthetic active radiation (PAR) can vary from <50 to 2000 µmol s^−1^ m^−2^ depending on cloud cover, and the total sum of PAR can vary from 2–65 mol m^−2^ day^−1^ (Hansen and Grimnes 2015). In Norway, it may be necessary to shelter the bioreactor from winter weather, and cultivate algae inside a greenhouse, which reduces the PAR with about ~50 % (Skov 1989). During the night, a significant amount of the biomass produced during the day is lost through respiration. Starch, which is the main storage carbon product in *Chlorella,* is a direct effect of photosynthesis and can be reduced by 50 % (Branyikova et al. 2011; Hirokawa et al. 1982). Apart from the length of the dark period, the biomass loss is also affected by temperature, growth phase and biochemical composition (Doucha and Livansky 2006; Han et al. 2013; Ogbonna and Tanaka 1996; Torzillo et al. 1991).

Temperature is affecting the biomass productivity and nutrient assimilation through the rates and nature of cellular metabolism (Richmond 1999). Controlling the temperature of the algal culture may therefore increase the biomass productivity and nutrient removal significantly. Han et al. (2013) showed that the biomass productivity of *Chlorella pyrenoidosa* increased by 37.8 % when the culture was heated during day. Vonshak et al. (2001) has shown that heating the culture for a shorter time period in the morning is sufficient for avoiding photoinhibition, and the biomass yield is increased. Some species, such as the diatom *Phaeodactylum tricornutum* needs to have a fully controlled temperature as the growth rate abruptly decreases above 20–23 °C (Benavides et al. 2013). Algal assimilation of ammonium (NH_4_-N), the major nitrogen compound in SL, is considered to be independent of temperature and will therefore depend on the growth of the selected microalgae (Reay et al. 1999). Phosphate (PO_4_-P) removal can be affected by both light and temperature (Hessen et al. 2002; Powell et al. 2009).

The aim of this study was to quantify the effect of temperature control on biomass production and nutrient removal at natural light, of a mesophilic C*hlorella* strain. This was elucidated in five experiments from May to September with different levels of temperature control: no-, partly- and full-temperature control and by a controlled day temperature combined with low (uncontrolled) night temperature.

## Methods

### Algae cultivation

The strain NIVA-137 *Chlorella sp*. was received from the Norwegian Institute for Water Research culture collection in Oslo, Norway, and has proven to grow at high rate in SL (Åkerström et al. 2014). SL was collected at a municipal WWTP (41,000 population equivalents), Nordre Follo Renseanlegg, located at Vinterbro, Norway. The sludge had been through anaerobic thermophilic digestion at a minimum temperature of 55 °C. SL was collected at the dewatering centrifuge where the dewatering process was aided by a cationic polymer and tap water. The SL was diluted with tap water to 25 % v/v due to a high total suspended solids (TSS) content. The composition of SL after dilution is shown in Table 1.

**Table 1:** The composition of SL after dilution with tap water to 25 % v/v

Compound (mg L^−1^)	
TN	304 ± 6.1
NH_4_-N	253 ± 20
NO_3_-N^a^	6.0
NO_2_-N^a^	0.6
TP	33.3 ± 1.52
PO_4_-P	23.0 ± 1.23
K	26.0 ± 1.0
Ca	22.5 ± 0.5
S	16.8 ± 1.04
Mg	3.7 ± 0.08
Fe	3.1 ± 0.17
Mn	0.3 ± 0.02
Cu	0.3 ± 0.25
Zn	0.7 ± 0.68
Al	75.0 ± 2.00
TSS	748 ± 198
pH	7.9 ± 0.2
COD	1360 ± 60

*TN* total nitrogen, *TP* total phosphorus

^a^Measured after sampling at the WWTP while the other parameters were measured prior to use

**Fig. 1:**
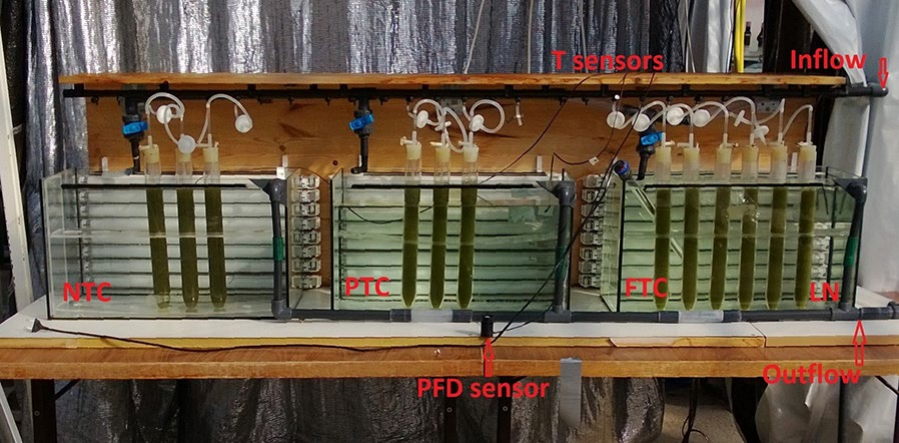
The experimental set-up showing the different levels of temperature control; no temperature control (NTC) at which the tubes were placed in air, partly temperature control (PTC) at which the tubes were placed in water with no heating or cooling, full temperature control (FTC) of 25 °C and one treatment that was kept in the thermostatic water batch during day (08:00–20:00) and was placed in air during night (LN)

*Chlorella* sp. was grown as batch cultures in glass tubes (3.5 cm inner diameter, 40 cm height) inside a greenhouse where the bioreactors were facing south. The greenhouse was located in Ås, Norway, (59°40′N 10°50′E). The first experiment was conducted in May, the second in June, experiments 3 and 4 in July and experiment 5 in September. The levels of temperature control were; (1) no temperature control (NTC), (2) partly temperature control (PTC), (3) full temperature control (FTC) and (4) a controlled day temperature combined with a low night temperature (LN). At NTC the tubes were placed in air, at PTC the tubes were placed in water and at FTC the tubes were placed in a thermostatic water bath that was controlled at 25 ± 1 °C. At LN the tubes were placed in the thermostatic water bath during day (08:00–20:00) and were moved manually to be placed in air during night (20:00–08:00). The experimental set-up is shown in Fig. 1.

Water baths used for temperature control had no impact on effects of irradiance. This was confirmed by measuring irradiance in empty glass tubes, placed in water or placed in air, at three irradiance levels. A paired student t-test showed that tubes placed in water and tubes placed in air received similar irradiance levels (p = 0.342, df = 107).

PAR was measured as photon flux density (PFD) inside the greenhouse next to the culture tubes using a quantum sensor (400–700 nm) LI-COR model LI-190SA instrument (LI-COR, Lincoln, NE, USA). The daily light integral (DLI) is the sum of photons received during the day and has the unit mol day^−1^ m^−2^ (Mortensen and Gislerod 2014). CO_2_ enriched air, 3 ± 0.2 % CO_2_, was provided to the cultures via a capillary tube after filter sterilization (Acro^®^ 37 TF Vent Devices, 0.2 µm) and the CO_2_ was measured by a gas analyzer (WHA-4, PP systems). The environmental parameters; PFD, temperature and CO_2_ were logged with a modified Campell CR10 logging and controlling system (Campell Scientific, Inc.) that also controlled the CO_2_ level. The inoculums were prepared using diluted SL and growing them inside the greenhouse at each level of temperature control. The start concentration was 20 × 10^6^ cells mL^−1^ and the cultivation period was 6 and 9 days for experiment 1 and 2, respectively. For the other experiments, the start concentration was 95 × 10^6^ cells mL^−1^ and the cultivation period was 3 days. The higher cell concentration and shorter time period was chosen to compare the cultures in the linear growth phase (Richmond 1999).

Every 12th hour (08:00 and 20:00) were pH, cell number and TSS measured. The reduction in NH_4_-N and PO_4_-P was measured every 12th hour during experiment 2 but was otherwise measured at the end of the cultivation period. Total nitrogen (TN) and phosphorus (TP) in the algal biomass was analyzed every 12th hour during experiments 3 and 4. Starch was analyzed every 12th hour for all experiments apart from experiment 1.

### Analytical methods

SL was analyzed for chemical oxygen demand (COD), total nitrogen (TN), total phosphorus (TP), ammonium (NH_4_-N) and phosphate (PO_4_-P) by using Hach-Lange kits, Hach DR 2800 Spectrometer and thermostat 2T 200 (Hach Lange, Germany). Inductively Coupled Plasma Optical Emission Spectrometer(ICP) (Optima 5300 DV, Perkin Elmer, USA) was used to analyze for sulfur (S), potassium (K), calcium (Ca), iron (Fe), magnesium (Mg), manganese (Mn), zinc (Zn), copper (Cu), and aluminum (Al). Before ICP measurements the samples were decomposed by addition of HNO_3_ to 10 % v/v and heated by ultraclave (ultraClave III, MLS, Leutkirch) to 250 °C for 1.5 h. The pH was measured by a calibrated pH-meter, Orion Aplus tm (Thermo electron corporation, USA). NH_4_-N and PO_4_-P were measured according to Norwegian Standard methods (NS 4746 and NS 4724, respectively). The nutrient removal rates were calculated as (mg nutrient L^−1^ day^−1^) = (C_1_ − C_2_) / (t_2_ − t_1_) were C_1_ and C_2_ are the nutrient concentrations at the start (t_1_) and the end (t_2_) of the cultivation period, respectively.

TSS was measured by filtering 0.3–0.5 mL algae culture diluted into 10 mL Milli-Q water (Milli-Q Synthesis A10, Millipore Corporation, USA) onto glass fiber filters (Whatman GF/F). The filters were further washed with 20 mL Milli-Q water, and then dried at 105 °C for 3 h. The filters were cooled in a desiccator before weighing by a Mettler Toledo XP6 microbalance (Mettler-Toledo Inc., USA). Triplicate TSS samples were carried out for each culture tube. Due to different length of the cultivation period the net biomass productivity was calculated by dividing the biomass obtained during the cultivation period by the number of cultivation days: (g L^−1^ day^−1^) = (TSS_2_ − TSS_1_)/ (t_2_ − t_1_), where TSS_1_ and TSS_2_ are the TSS concentrations at the start (t_1_) and the last evening of the cultivation period (t_2_), respectively. The day productivity is the increase in biomass from the morning (08:00) until the evening (20:00) whereas the biomass loss is the reduction from the evening (20:00) until the morning (08:00).

Cells were counted using a cell counter (Beckman Coulter Multisizer 4, Beckman Coulter, Inc.) with an aperture of 2 µm. The mass per cell was calculated by dividing the TSS content (mg L^−1^) by the number of cells (10^6^ mL^−1^) multiplied with 1000 to present the values as microgram (µg) per 10^6^ cells. The number of cell doublings (*k*) during night (per 12 h) were calculated as *k* = ( ln *N*_*t*_ − ln *N*_0_)/0.6931 were N is the number of cells at time t (Andersen 2005).

The starch content in the biomass (measured as TSS) was analyzed by harvesting 1–2 mL algae suspension by centrifugation at 14,000 rpm for 10 min (Eppendorf 5417 R). Equal portions of cell disruption beads 0.5 mm (Scientific Industries S1-BG 05) and 0.1 mm (Scientific Industries S1-BG 01) were added until a total volume of 0.5 mL was achieved. Methanol was added and the cells were broken by a Mixer Mill Type MM 301 (Retch, Germany) for 10 min at 30 Hz. The chlorophyll extract was separated by centrifugation at 14,000 rpm for 10 min. The remaining pellet was used for starch analyses. The total starch content was determined by an enzymatic procedure using α-amylase and amyloglucosidase following the protocol in the starch assay Megazyme AOAC Method 996.11 AACC (Megazyme International, Ireland). The method was modified to allow for smaller samples.

To measure TP and TN in the biomass a volume of 10 mL algae suspension was harvested by centrifugation at 4000 rpm for 10 min (Eppendorf Centrifuge 5810). The supernatant was removed and the cell pellet was washed three times by re-suspension in Milli-Q water (Milli-Q Synthesis A10, Millipore Corporation, USA) and centrifugation at 4000 rpm for 5 min. After a final re-suspension to 10 mL, TN was measured as described by Norwegian Standard (NS 4743). TP was measured by using the same methods as for the SL. The percentage TN and TP in the TSS samples were calculated by TN or TP (g L^−1^)/TSS (g L^−1^) × 100. The proportion of removed ammonium and phosphate that was incorporated into the biomass was calculated as: [removed NH_4_-N or PO_4_-P]/[TN or TP in the biomass] × 100.

General regression models were made for the response variables: day productivity, biomass loss, and starch content. When the level of temperature control was a significant factor, a general linear model (GLM) was carried out with Tukey’s comparison. ANOVA and Tukey’s ad hoc test was used when comparing the treatments at one sampling point. Statistical tests were executed in Minitab 16 software and graphs were made in SigmaPlot 10.0.

## Results and discussion

### Temperature and light conditions

**Fig. 2:**
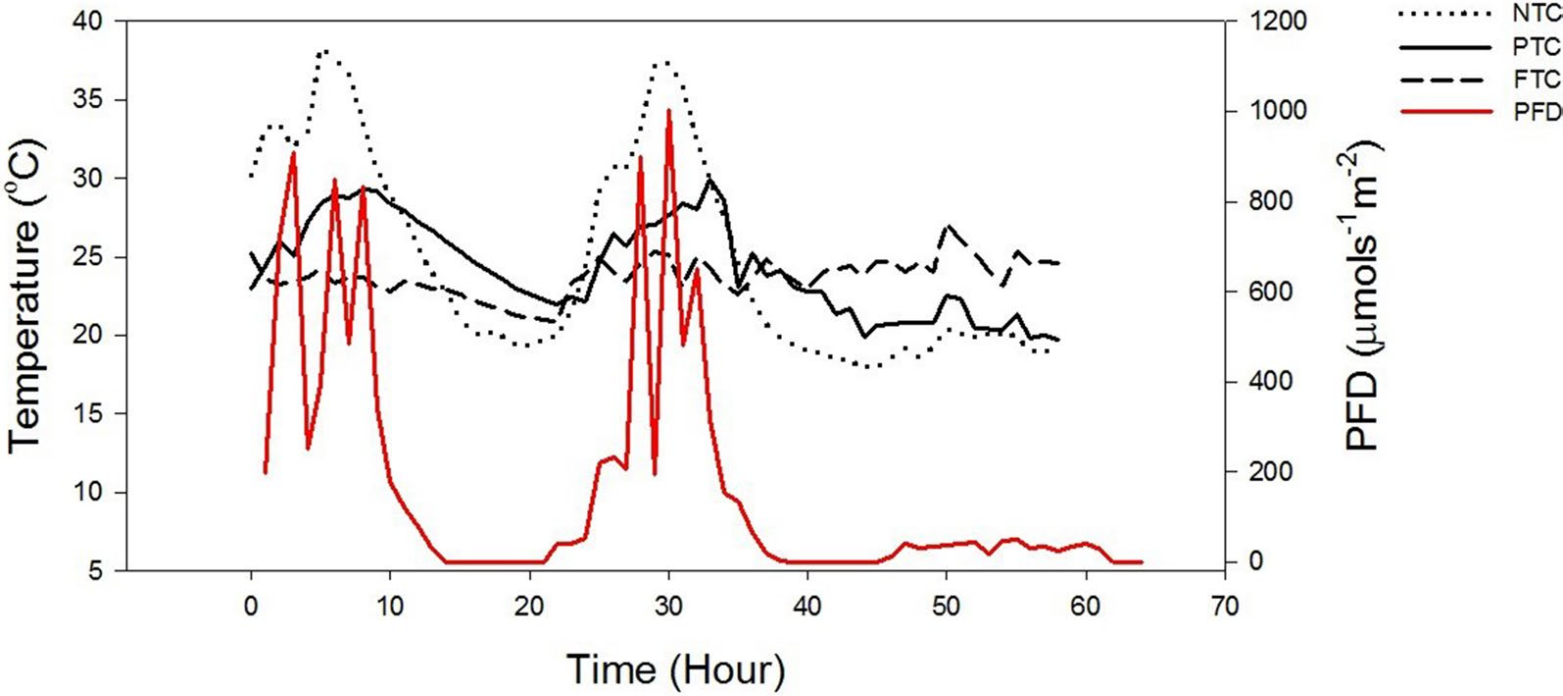
Photon flux density (PFD) and Temperature of *Chlorella* sp. cultures at no (NTC), partly (PTC) and full (FTC) temperature control during 26–28 July (experiment 4)

The average DLI varied from 9–22 mol day^−1^ m^−2^ during the five experiments (Table 2). The average temperature varied from 17.7–24.9 and 16.7–24.5 °C at NTC and PTC, respectively. At LN, the average temperatures were 18.8 and 20.6 °C at experiment 1 and 2, respectively. At clear sky, PFD’s of 800–1000 µmol s^−1^ m^−2^ were reached inside the greenhouse and the temperature exceeded >30 °C at NTC (Fig. 2). The peak in temperature either coincided with the peak in PFD or had a delay of 1–2 h. The maximum temperature reach was 38.1 °C at the fourth experiment. At PTC the temperature rarely exceeded 30 °C. During night, the temperature dropped to ~10 °C at NTC during the first two experiments and to 15–20 °C in the other three experiments. The night temperature was generally ~2 degrees higher at PTC compared to NTC. The maximum and minimum temperatures lasted for a period of 1–3 h resulting in less variation between the average day and night temperatures. The heating system at FTC was not always sufficient for keeping the temperature at 25 °C during night, which resulted in lower night temperature (23.2–23.9 °C) in experiments 1, 3 and 4.

**Table 2:** The average daily light integral (DLI), average temperature (T) °C, day- (DT) and night temperatures (NT) at five experiments executed from May to September

Exp. (month)	1 (May)			2 (June)			3 (July)			4 (July)			5 (September)		
DLI (mol day^−1^m^−2^)	9 ± 4.5			22 ± 4.9			10 ± 4.1			12 ± 9.2			13 ± 3.6		
	ΔT	DT	NT	ΔT	DT	NT	ΔT	DT	NT	ΔT	DT	NT	ΔT	DT	NT
NTC	17.7 (±6.5)	20.9 (±5.9)	12.1 (±4.3)	22.6 (±8.2)	29.6 (±4.6)	15.3 (±3.2)	23.9 (±5.9)	27.4 (±3.4)	19.5 (±1.7)	24.9 (±6.5)	28.2 (±6.6)	20.0 (±1.5)	21.1 (±5.1)	25. 1 (±3.7)	17.3 (±1.4)
PTC	16.7 (±2.8)	17.7 (±2.8)	15.5 (±2.4)	23.4 (±4.5)	23.0 (±7.6)	17.7 (±2.0)	23.0 (±2.2)	23.8 (±2.3)	21.9 (±1.5)	24.5 (±3.0)	25. 5 (±3.7)	22.9 (±1.2)	22.2 (±2.4)	23.2 (±1.4)	21.0 (±.7)
FTC	24.2 (±1.1)	24.7 (±0.8)	23.2 (±0.64)	24.9 (±0.6)	24.9 (±0.7)	25.0 (±0.2)	24.0 (±2.5)	24.0 (±0.6)	23.9 (±0.4)	23.7 (±1.7)	24.2 (±1.2)	23.2 (±1.4)	25.2 (±0.5)	25.2 (±0.9)	25.2 (±0.1)
LN	18.8 (±7.3)	24.7 (±0.8)	12.1 (±4.3)	20.6 (±5.9)	24.9 (±0.3)	15.3 (±1.3)

### Biomass productivity

#### Net biomass productivity

The biomass productivity at FTC varied from 0.26–0.46 g L^−1^ day^−1^ and was higher or equal to the other levels of temperature control (Table 3). At experiment 1 in May, the DLI was low, the productivity was reduced (0.18–0.20 g L^−1^ day^−1^) at NTC, PTC, and LN compared to FTC (0.26 g L^−1^ day^−1^). At the second experiment in June, the average DLI was high (22 mol day^−1^ m^−2^) and the productivity was similar at NTC, PTC and FTC (0.40–0.43 g L^−1^ day^−1^) while significantly reduced at LN (0.33 g L^−1^ day^−1^). The reduced productivity at LN was assumingly due to the lower average temperature of 20.6 °C, compared to NTC and PTC (22.6 and 23.4 °C) as the night temperature was equal to NTC and the day temperature equal to FTC. At the third experiment in July, NTC, PTC and FTC had similar biomass productivities (0.35–0.38 g L^−1^ day^−1^) and the average temperatures were also similar. At the forth experiment in July, the biomass productivity was lower at NTC (0.37 g L^−1^ day^−1^) compared to PTC and FTC (0.41 and 0.46 g L^−1^ day^−1^, respectively). The average temperatures were similar but the reduction at NTC was probably due to high day temperatures that exceeded 35 °C the first 2 days of cultivation and remained >30 °C for the main part of the day (Fig. 2). For the fifth experiment in September, the productivity was significantly higher at FTC (0.44 g L^−1^ day^−1^) compared to NTC and PTC (0.27 and 0.26 g L^−1^ day^−1^, respectively). At this experiment, the DLI was higher than in experiments 3 and 4 but the photoperiod was shorter which resulted in lower average temperatures (21.1 and 22.2 °C at NTC and PTC, respectively). The results imply that the average temperature is of more importance than the day or night temperature range. This effect is similar to higher plants regarding both yield and photosynthesis (Dekoning 1988; Huckstadt et al. 2013). In conclusion, the productivity was reduced by 23 and 39 % when the average temperature was <22 °C, compared to a controlled temperature of 24–25 °C. At experiment 4, the productivity was reduced by 20 % compared to at FTC, due to supra-optimal day temperatures that exceeded 30 °C the main part of the day at NTC.

**Table 3:** Biomass productivity (g L^−1^ day^−1^) and cell number of *Chlorella* sp. at different levels of temperature (T) control: no (NTC), partly (PTC), and full temperature control (FTC) and at a controlled day temperature combined with low night temperature (LN)

	DLI (mol day^−1^ m^−2^)				
	9 ± 4.5	22 ± 4.9	10 ± 4.1	12 ± 9.2	13 ± 3.6
g L^−1^ day^−1^					
NTC^α^	0.20^b^ (±0.01)	0.41^a^ (±0.01)	0.38^a^ (±0.05)	0.37^b^ (±0.02)	0.27^b^ (±0.06)
PTC^α^	0.18^b^ (±0.01)	0.40^a^ (±0.02)	0.35^a^ (±0.05)	0.41^ab^ (±0.03)	0.26^b^ (±0.04)
FTC^α^	0.26^a^ (±0.01)	0.43^a^ (±0.05)	0.35^a^ (±0.02)	0.46^a^ (±0.01)	0.44^a^ (±0.04)
LN^α^	0.19^b^ (±0.01)	0.33^b^ (±0.00)			
No. cells (10^6^ mL^−1^)					
NTC	177 (±3)	224 (±16)	202 (±5)	222 (±11)	253 (±16)
PTC	194 (±12)	287 (±5)	207 (±7)	330 (±13)	288 (±5)
FTC	204 (±25)	248 (±13)	205 (±11)	352 (±24)	191 (±33)
LN	179 (±43)	289 (±18)			

Results from five experiments conducted inside a greenhouse at natural light and different average daily light integral (DLI). Average values and standard deviation in parenthesis

^α^A different letter denotes significant differences (p < 0.05), between the levels of T control

At FTC the productivity was similar at average DLI’s 12, 13 and 22 mol day^−1^ m^−2^ (Table 3) and is in comparison to Sasi et al. (2011) that showed that the growth rate of *Chlorella vulgaris* increased according to a Monod equation with increasing PAR. At a typical light response curve, the photosynthesis rate increases with increasing light intensity until the photosynthetic machinery is no longer limited by light but by downstream processes. The photosynthesis rate is at its maximum and excess light is dissipated as heat. The productivity values of ~0.45 g L^−1^ day^−1^ is comparable to the maximum productivity obtained in a previous study conducted with the same strain, at continuous lighting of 115 µmol^−1^ m^−2^ and in both diluted SL and in a balanced mineral medium (Åkerström et al. 2014). The productivity is also affected by particles in the SL and cell density. In batch cultures, the cell density increases over time and thereby decreases the amount of light received per cell. At a certain cell density all incoming photons are reduced by the algae cells and the biomass will increase linearly, while at a lower cell density the culture can grow exponentially (Richmond 2004). In this study, the cultures were mainly in the linear growth phase and can therefore be regarded as light saturated at ≥12 mol day^−1^ m^−2^. Sandnes et al. (2005) showed a linear relationship with PAR and the productivity of *Nannochloropsis oceanica* in the linear growth phase and the highest productivity of 0.7 g L^−1^ day^−1^ was obtained at a continuous lighting of 1030 µmol s^−1^ m^−2^. This level of light corresponds to 89 mol day^−1^ m^−2^ and such levels is not possible when using natural light. Apart from cell density the light path (LP), i.e. the diameter of the photobioreactor or the depth of the culture in a pond or raceway, is important for the productivity as it also decides the amount of light received per cell in a unit of time. In a study by Munkel et al. (2013), that grew *Chlorella vulgaris* in an outdoor biofence with a similar LP, a productivity of 0.64 g L^−1^ day^−1^ was obtained but at nitrogen limitation. Other productivities using natural light has been reported by Zhou et al. (2013) and Feng et al. (2011) that had productivities of 154.48 and 58.4 mg L^−1^ day^−1^ of *Chlorella* sp. in outdoor flat panel bioreactors but at LP’s of 22 and 40 cm, respectively. Osendeko and Pittman (2014) had a productivity of 0.4 g L^−1^ day^−1^ during summer when growing *Parachlorella husii* in diluted sludge liquor from dewatering activated sludge (prior digestion) inside a glasshouse with a LP of 10 cm.

**Fig. 3:**
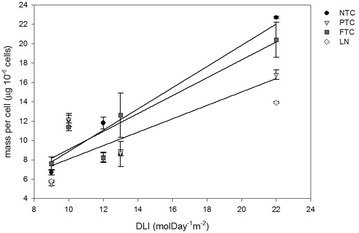
Mass per cell (µg 10^−6^ cells) the last evening of cultivation as a function of average daily light integral (DLI) during the experiments

**Fig. 4:**
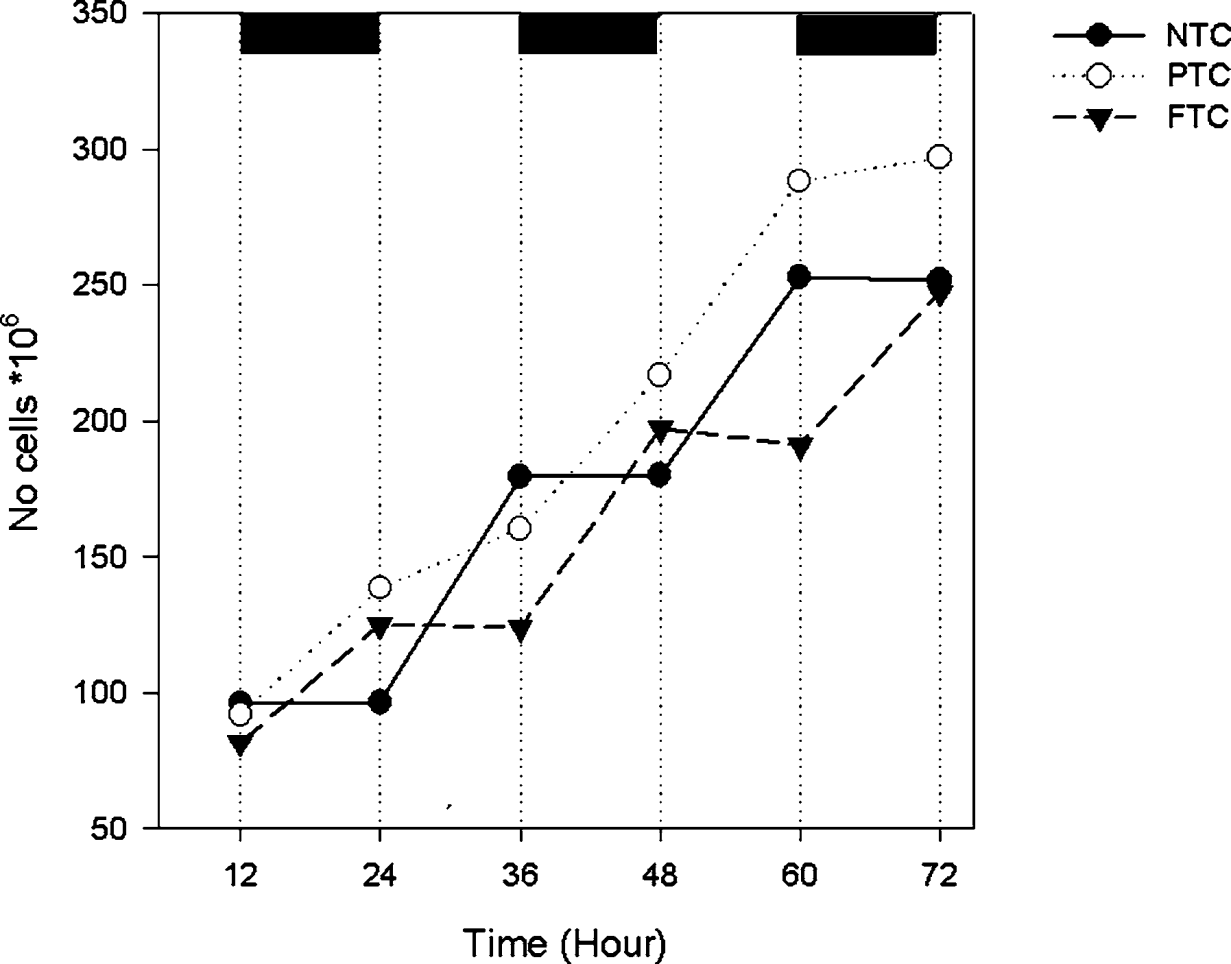
Number of cells as a function of time in cultures at no temperature control (NTC), partly temperature control (PTC) and full temperature control (FTC) during experiment 5

Higher biomass productivity did not always result in a higher cell number (Table 3) but was due to a higher mass per cell. Figure 3 shows the mass per cell the last evening of the cultivation period for all five experiments. It increased with increasing DLI from ~5–7 µg 10^−6^ cells^−1^ to 22.7, 16.8 and 20.4 µg 10^−6^ cells^−1^ at NTC, PTC and FTC, respectively. It is noticeable that FTC had lower cell number than NTC and PTC at the fifth experiment (Table 3). This was due to cells divided during day at NTC and PTC, instead of during night as in FTC (Fig. 4). The cell division stops at a certain lower temperature due to the inability to perform the necessary enzymatic reactions (Ahlgren 1987). The night temperature was not lower than in the other experiments but the dark period was longer which means a prolonged period at low temperature. This may have induced cell division during day and resulted in reduced productivity, as this is an energy demanding process.

#### Day productivity

**Fig. 5:**
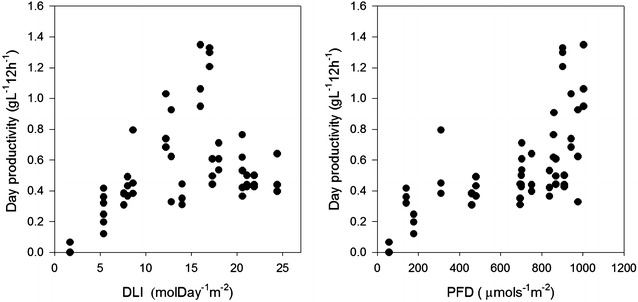
The day productivity (08:00–20:00) as a function of the daily light integral (DLI) or the maximum photon flux density (PFD) of cultures at full temperature control (FTC)

The productivity during day (08:00–20:00) varied from <0.1 g L^−1^ 12 h^−1^ to 1.4 g L^−1^ 12 h^−1^ and was on average 0.55 g L^−1^ 12 h^−1^ at FTC and 0.48 g L^−1^ 12 h^−1^ at NTC and PTC (p = 0.001). At LN the average day productivity was 0.33 g L^−1^ 12 h^−1^. At FTC, the day productivities increased linearly with DLI to 10 mol day^−1^ m^−2^ (Fig. 5). Above 12 mol day^−1^ m^−2^ there was large variations but if removing values >0.8 g L^−1^ 2 h^−1^ a sigmoid curve is obtained (R^2^ = 0.72). The maximum PFD was also of significance for the day productivity (p = 0.001) and the highest productivities were obtained at PFD’s > 900 µmol s^−1^ m^−2^ (Fig. 5). The peak in irradiance lasted for only a few hours but during that time, light can penetrate deeper into the culture and is thereby increasing the amount of light received per cell. The highest day productivities, >0.8 g L^−1^ 12 h^−1^, were obtained at cell densities <150 × 10^6^ cells mL^−1^ and TSS values <2 g L^−1^ and was thus a combination of received light and cell density. However, lowering the cell density in a large-scale system may not be practical as harvesting costs may increase and the culture is more exposed to photoinhibition (Sandnes et al. 2005).

**Fig. 6:**
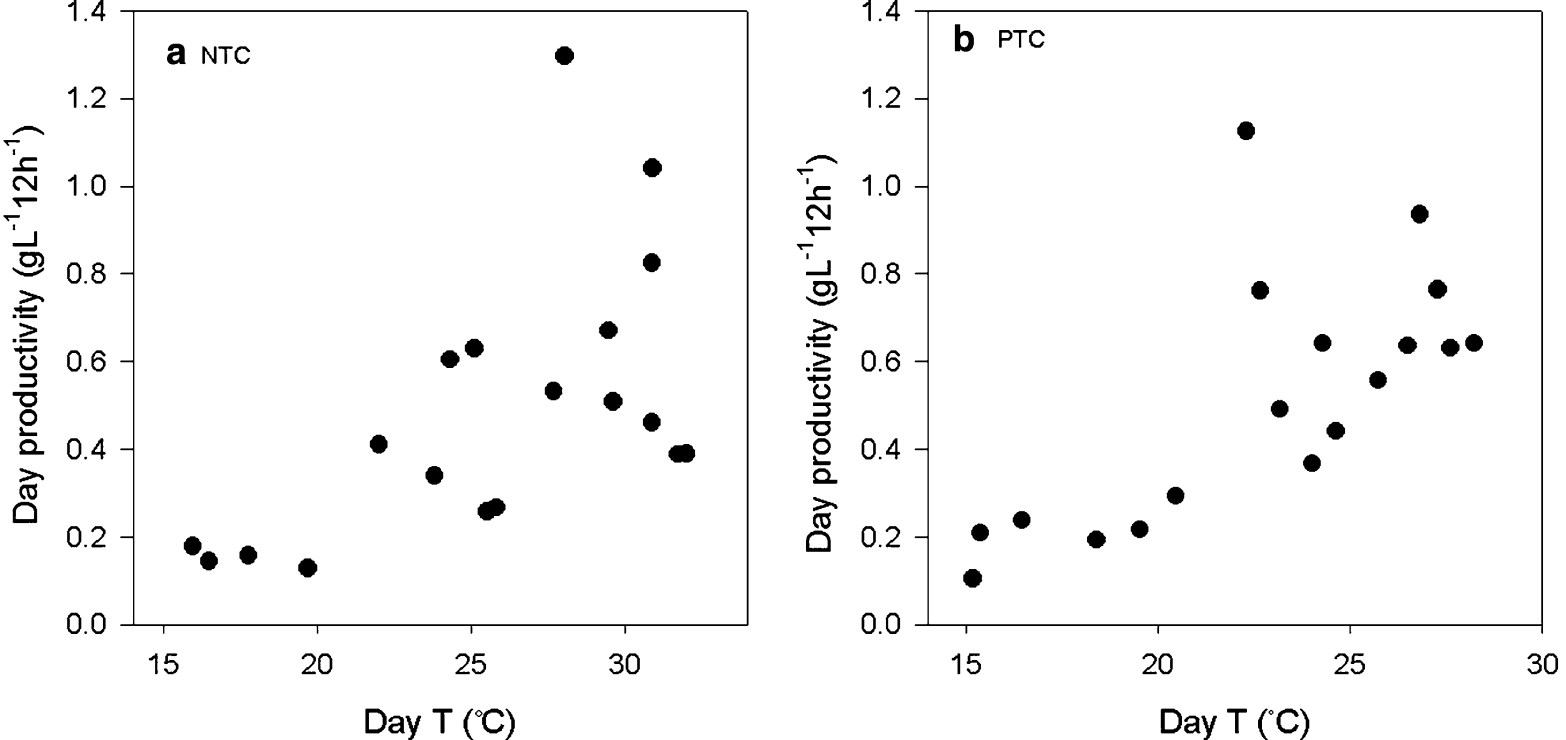
Day productivity (08:00–20:00) of *Chlorella sp*. grown at **a** no temperature control (NTC) and **b** partly temperature control (PTC)

At NTC and PTC the day productivity was also dependent on temperature, (in addition to irradiance), which is an indirect effect of PFD (p = 0.003 and 0.002, respectively). At average day temperatures, <22 °C the productivity was consistently low (Fig. 6). At temperatures >22 °C, there was large variations in the productivity (0.26–1.30 g L^−1^12 h^−1^). The highest productivities were obtained in a range from 22–31 °C showing that *Chlorella sp*. has a wide plateau for in optimal temperature. The excitation of PSII in photosynthesis is not light dependent but downstream processes are as they involves enzymatic processes (Raven and Geider 1988). Therefore, the optimal temperature increases with PFD, and the point of light saturation increases with temperature (Bouterfas et al. 2002). In an algal cultivation system that uses solar irradiance, temperature may thus be controlled within the optimal temperature range as a function of irradiance. In temperate regions such as Norway, heating the culture to avoid suboptimal temperatures in periods with low PFD’s or short photoperiods would greatly increase the biomass production while cooling of the culture may be necessary only occasionally.

#### Nightly biomass loss

The average reduction in biomass during night was 0.13, 0.08 and 0.09 g L^−1^ at FTC, NTC and PTC, respectively (data not shown). At LN the average reduction was 0.01 g L^−1^. These values correspond to a percentage reduction of 19, 17, 24 and 3 % at NTC, PTC, FTC and LN, respectively. The percentage biomass loss is rather high when compared to other studies that has reported reductions of 3–21 % with 7–10 % being the most common values (Benavides et al. 2013; Han et al. 2013; Ogbonna and Tanaka 1996; Torzillo et al. 1991). The biomass loss during night increased with night temperature, (p = 0.023) but the variations were large (R^2^ = 0.28, obtained from general regression analysis). This may partly be due to miss match in the sampling times as the cultures were exposed to some light (<40 µmol s^−1^ m^−1^) before and after sampling (~1 h) and that the biomass loss is also affected by the age of the culture (Ogbonna and Tanaka 1996). However, our results show that a higher biomass loss, i.e. cell respiration is will result in higher day productivity and a higher net productivity. Too low night temperature is therefore not recommended.

#### Starch content

The percentage of starch in the biomass varied from 4.1–11.7 % at the end of the night (08:00) to 15.7–37.8 % at the end of the day (20:00) (Table 4). In general, the starch content was significantly lower at NTC compared to FTC at the end of the day (p = 0.037) while there was no significant difference between the levels of temperature control at the end of night (p > 0.05). The reduced starch content at NTC was assumingly due to a higher day temperature. Our previous results (unpublished) has shown that the starch content is reduced at 30 °C compared to 25 °C. Other studies have shown that starch is degraded at higher temperatures while the sucrose content increases or the starch to lipid ratio is changed (Converti et al. 2009; Nakamura and Miyachi 1982).

**Table 4:** The percentage starch content at 08:00 and 20:00 O’clock at different levels of temperature control; no (NTC), partly (PTC), full temperature control (FTC) and controlled day temperature combined with low night temperature (LN)

Experiment	1	2	3	4	5
NTC_08:00_	∆	11.7 ± 2.6	5.5 ± 0.5	6.3 ± 2.2	7.3 ± 2.2
PTC_08:00_	∆	9.1 ± 0.7	5.8 ± 0.3	5.0 ± 0.3	6.3 ± 1.7
FTC_08:00_	∆	9.5 ± 1.5	6.4 ± 0.9	4.1 ± 0.6	8.2 ± 2.3
LN_08:00_	∆	9.0 ± 1.3			
NTC_20:00_	∆	15.7 ± 3.2	22.2 ± 4.1	18.9 ± 2.7	30.6 ± 5.2
PTC_20:00_	∆	17.3 ± 2.2	28.8 ± 1.7	21.5 ± 4.2	36.9 ± 1.6
FTC_20:00_	∆	17.7 ± 3.0	28.7 ± 2.9	23.7 ± 7.4	37.8 ± 7.9
LN_20:00_	∆	15.9 ± 4.4			

∆ not measured

**Fig. 7:**
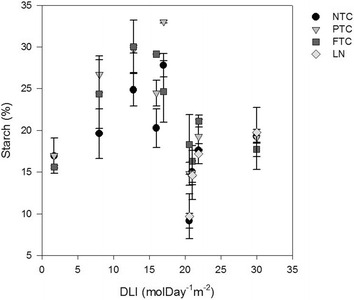
Cellular percentage starch content at the end of day (20:00) as a function of the daily light integral (DLI)

**Fig. 8:**
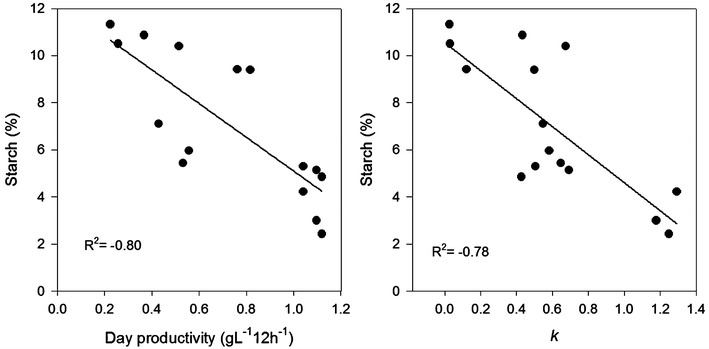
Cellular percentage starch content at the end of night (08:00) as a function of the productivity the previous day (*left*) and as the number of doublings during night (*right*) for experiments 3–5 (July–September)

The highest starch content, measured at the end of the day (>24 %) was obtained at 8–17 mol day^−1^ m^−2^ (Fig. 7) and does not correlate with mass/cell that increased linearly with PAR (Fig. 3). The lack of correlation indicates allocation of starch into other structural changes or production of another carbon storage product. High light adaptations is an increase in cell volume, carbon content and the number and density of thylakoid membranes (Berner et al. 1989; Falkowski and Owens 1980). A larger cell volume implies an increased synthesis of cell wall and membrane constituents. Although starch is the general carbon storage product for green algae some strains of *Chlorella* accumulates lipids which can increase with increasing amount of light (Sasi et al. 2011). The starch content at the end of the night was negatively correlated with the productivity on the previous day (p = 0.001) and with the number of doublings during the night (p = 0.001) as shown in Fig. 8. It implies that after high day productivity more cells can divide during night that result in a lower starch content in the morning. The results also implies that for the purpose of anaerobic digestion, harvesting should be carried out at the end of day, when the starch content is at its highest (Table 4).

**Fig. 9:**
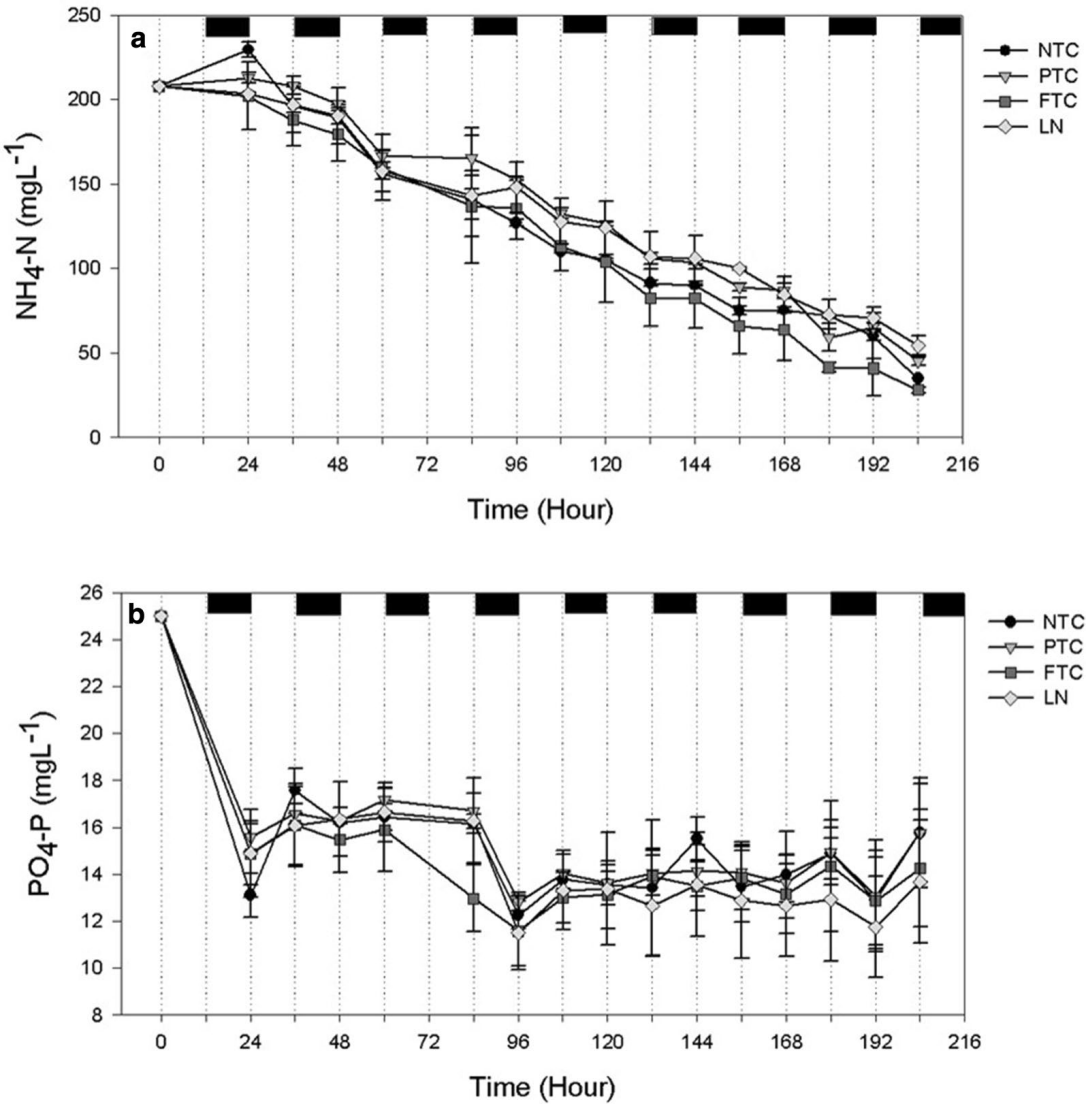
Reduction in **a** ammonium (NH_4_-N mg L^−1^) and **b** phosphate (PO_4_-P mg L^−1^) as a function of time in diluted sludge liquor during experiment 2 at no (NTC), partly (PTC), full temperature control (FTC) and at a controlled day temperature combined with a low night temperature (LN). Dark periods are marked as *black*

### Removal of ammonium and phosphate

**Fig. 10:**
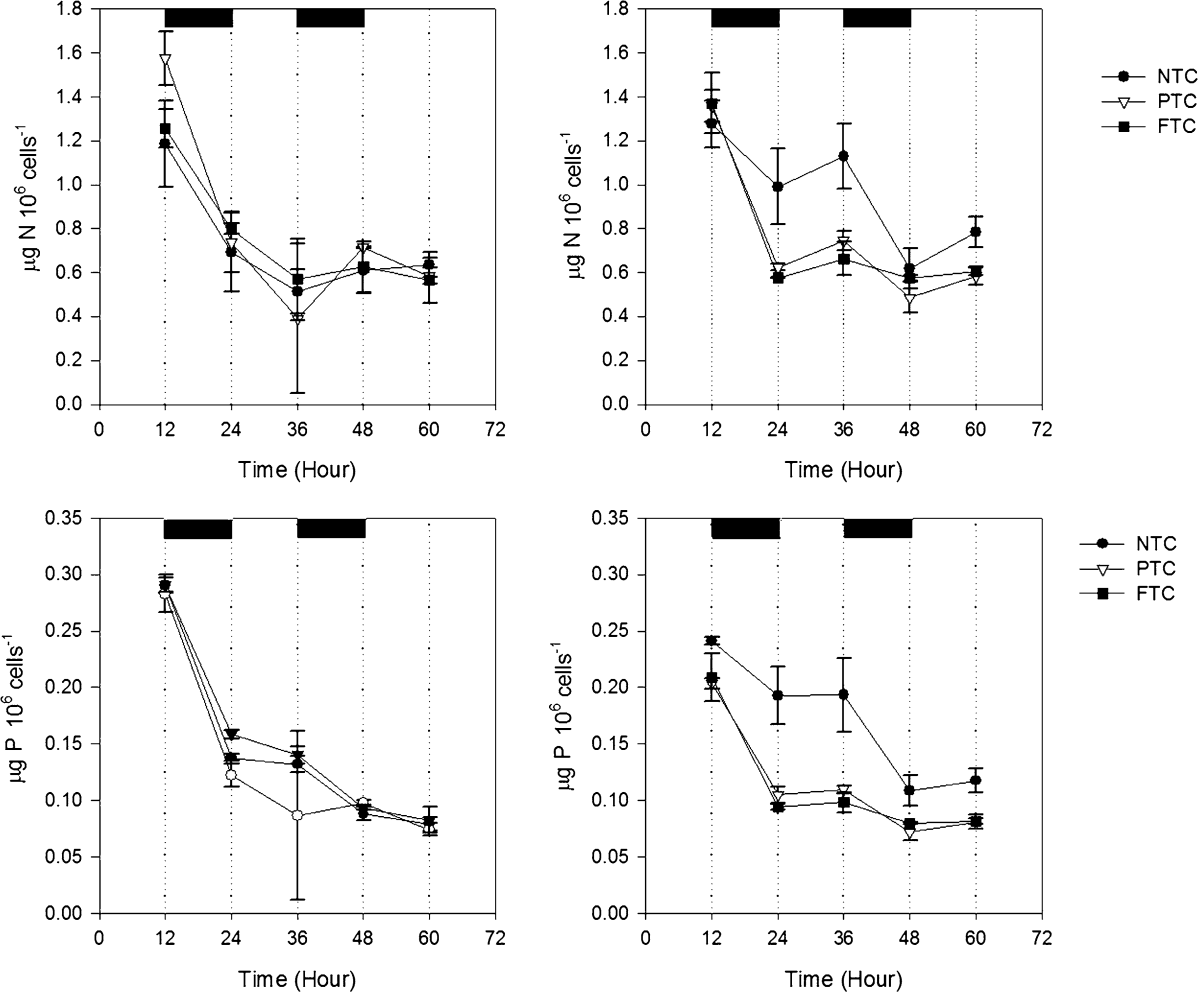
Cellular contents of N and P (µg 10^−6^ cells) of *Chlorella sp*. as a function of time grown at no (NTC), partly (PTC), and full temperature control (FTC) during experiment 3 (*left*) and experiment 4 (*right*). Dark periods are marked as black

The NH_4_-N removal rate varied from 12.7–35.0 mg L^−1^ day^−1^ (Table 5). The PO_4_-P removal rates varied from 1.3–3.8 mg PO_4_-P L^−1^ day^−1^ with no difference between the levels of temperature control. Nutrients are assimilated by individual cells and growth in terms of cell number is an important factor for nutrient removal. For algal nutrient removal in SL temperature control is mainly important as it affects the growth rate of the algae. The results showed that cell number and biomass productivity is not necessarily correlated since productivity is also an effect of mass/cell. When excluding the storage carbon compounds starch and lipids the N and P contents in the cell are almost constant (Scherholz and Curtis 2013). In the first two experiments, there was a small difference in removal of NH_4_-N as mg L^−1^ day^−1^. This was due to a similar cell number at the different levels of temperature control. The small differences in NH_4_-N removal rates at similar cell number shows that NH_4_-N removal was independent by temperature but was removed in proportions of availability (Reay et al. 1999). The largest difference in removal rates was obtained in experiment four (in July) in which it was significantly reduced at NTC compared to PTC and FTC and thereby correlated with a lower cell number at NTC. The lack of difference in NH_4_-N removal rates at experiment 5 despite differences in cell number may be due to that cells had divided at NTC and PTC but not started to assimilate nutrients (See “Net biomass productivity” in section). Figure 9 shows the reduction in NH_4_-N and PO_4_-P during experiment 2. NH_4_-N was removed during day and decreased more or less linearly during the cultivation period while PO_4_-P was removed during the first 24 h after which it fluctuated around the same value. The assimilation of nitrogen into protein requires both energy and a carbon skeleton and is therefore typically removed during day (Turpin 1991). The fluctuation of PO_4_-P concentration in the supernatant during the remaining cultivation period may be due to cellular leakage or by the transformation of other P compounds to PO_4_-P by bacteria (Zhao et al. 2012). The nutrient removal is affected by nutrient concentration and cultivation period and that can be explained by the Monod model; when batch cultures are used, algae grow at the expense of a nutrient element and the cellular quota decreases as the nutrient element in the medium decreases (Kim et al. 2013). The reduction in PO_4_-P during the first 24 h is assumingly due to surface adsorption to the cells, a none active biogenic uptake process and surface adsorption to the culture vessel rather that precipitation of P (Martinez et al. 2000; Sanuo-Wilhelmy et al. 2004; Xu et al. 2014). This was shown by a decrease in N and P per cell that decreased 2- to 2.5-fold after 3 days of cultivation (Fig. 10) and also explains the lower removal rates at experiments 1 and 2 by the longer cultivation period. At experiments 3 and 4, the removed NH_4_-N and PO_4_-P constituted of 89 ± 11 and 69 ± 15 % of the TN and TP in the biomass, respectively.

**Table 5:** Ammonium (NH_4_-N) and phosphate (PO_4_-P) removal rates (mg L^−1^ Day^−1^) at no (NTC), partly (PTC), and full temperature control (FTC) and at a controlled day temperature combined with low night temperature (LN)

	Level of T control	Experiment (number of days)				
		1 (6)	2 (9)	3 (3)	4 (3)	5 (3)
NH_4_-N (mg L^−1^ day^−1^)	NTC^α^	16.5^a^ (±1.6)	19.2^a^ (±0.0)	28.4^a^ (±5.5)	24.6^b^ (±0.2)	28.4^a^ (±5.5)
	PTC^α^	12.7^b^ (±0.2)	18.1^b^ (±0.3)	32.7^a^ (±0.6)	32.6^a^ (±1.3)	32.7^a^ (±0.6)
	FTC^α^	16.8^a^ (±0.9)	20.0^a^ (±0.2)	31.1^a^ (±2.8)	35.0^a^ (±1.7)	31.1^a^ (±2.8)
	LN^α^	15.1^ab^ (±0.8)	17.1^b^ (±0.6)			
PO_4_-P (mg L^−1^ day^−1^)	NTC^α^	2.4 (±0.1)	1.3 (±0.1)	2.9 (±0.6)	3.7 (±0.1)	2.3 (±0.1)
	PTC^α^	2.3 (±0.1)	1.3 (±0.1)	2.8 (±0.1)	3.8 (±0.1)	2.3 (±0.3)
	FTC^α^	2.3 (±0.4)	1.3 (±0.2)	2.9 (±0.1)	3.7 (±0.1)	2.6 (±0.2)
	LN^α^	2.3 (±0.2)	1.5 (±0.2)			

Average values and standard deviations in parenthesis, n = 3

^α^A different letter denotes significant differences (p < 0.05), between the levels of T control

## Conclusion

The results show that high biomass productivities and nutrient removal rates can be obtained at a wide range in temperature and irradiance. At daily light integrals of 12–22 mol day^−1^ m^−1^ growth was light saturated and the maximum net productivity (0.45 g L^−1^ day^−1^) was reached. *Chlorella* sp. showed a wide plateau in optimal temperature and within this range, the average temperature was more important for productivity than the day or night temperature range. Controlling the temperature at day combined with a night temperature following the ambient air was not sufficient for obtaining a high productivity due to a lower average temperature. At average temperatures <22 °C the productivity was reduced by 23 and 39 %, compared to the full temperature control cultures at 24–25 °C. For one experiment, the productivity was reduced by 20 % at no temperature control compared to full temperature control due to supra-optimal day temperatures (>32 °C). At the end of day, the starch content was reduced at no temperature control compared to full temperature control as an effect of higher day temperatures. The level of temperature control had no effect on PO_4_-P removal but had an effect on NH_4_-N removal at supra-optimal temperatures at which it was reduced.
